# Crystal structures of 6-cyclo­propyl-1,3-diphenylfulvene and 6-(2,3-di­meth­oxy­naphth­yl)-1,3-di­phenylfulvene

**DOI:** 10.1107/S2056989020006441

**Published:** 2020-05-22

**Authors:** Loren C. Brown, Scott T. Iacono, Gary J. Balaich

**Affiliations:** a Department of Chemistry & Chemistry Research Center, United States Air Force, Academy, Colorado Springs, CO 80840, USA

**Keywords:** crystal structure, fulvene, C—H⋯C ring inter­actions

## Abstract

The title compounds, 6-cyclo­propyl-1,3-diphenylfulvene, C_21_H_18_, [systematic name: 5-(cyclo­propyl­methyl­idene)-1,3-di­phen­yl­cyclo­penta-1,3-diene], **1**, and 6-(2,3-di­meth­oxy­naphth­yl)-1,3-diphenylfulvene, C_30_H_24_O_2_, {systematic name: 5-[(3,4-di­meth­oxy­naphthalen-2-yl)methyl­idene]-1,3-di­phenyl­cyclo­penta-1,3-di­ene}, **2**, were prepared from 1,3-di­phenyl­cyclo­penta­diene, pyrrolidine, and the corresponding aldehydes in an ethano­lic solution. Each structure crystallizes with one mol­ecule per asymmetric unit and exhibits the alternating short and long bond lengths typical of fulvenes. A network of C—H⋯C ring inter­actions as well as C—H⋯O inter­actions is observed, resulting in the compact packing found in each structure.

## Abstract 2

The title compounds were prepared from 1,3-di­phenyl­cyclo­penta­diene, pyrrolidine, and the corresponding aldehydes in an ethano­lic solution. Each compound crystallizes with one mol­ecule per asymmetric unit and exhibits the alternating short and long bond lengths typical of fulvenes. A network of C—H⋯C ring inter­actions as well as C—H⋯O inter­actions is observed, resulting in the compact packing found in each structure.

## Chemical context   

Penta­fulvenes are a unique class of cross-conjugated organic mol­ecules commonly synthesized using aldehyde and cyclo­penta­diene starting materials under a variety of conditions (Thiele, 1900 ▸; Stone & Little, 1984 ▸; Sieverding *et al.*, 2019 ▸). Substituted and highly colored penta­fulvenes are of particular inter­est because of their unique optical and thermal properties and for their potential use in electronic applications (Peloquin *et al.*, 2012 ▸; Godman *et al.*, 2016 ▸; Shurdha *et al.*, 2014 ▸). In synthetic organometallic chemistry, the fulvene unit is known to coordinate to metals, forming organometallic complexes of varying hapticity (Peloquin *et al.*, 2018 ▸; Ma *et al.*, 2011 ▸, 2012 ▸; Beckhaus, 2018 ▸). More recently, 1,3,6-tris­ubstituted fulvenes have been used as starting materials in the synthesis of bridged cyclo­penta­diene ligands and *ansa*-*Ln* complexes (Adas & Balaich, 2018 ▸). As a continuation of our work in this area, we report herein the crystal structures of 6-cyclo­propyl-1,3-diphenylfulvene, **1**, and 6-(2,3-di­meth­oxy­naphth­yl)-1,3-diphenylfulvene, **2**.
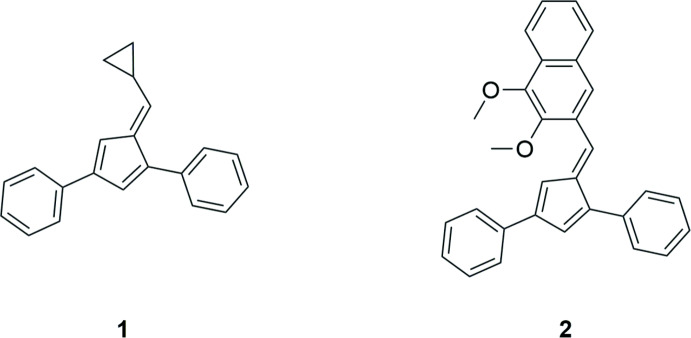


## Structural commentary   

**Figure 1:**
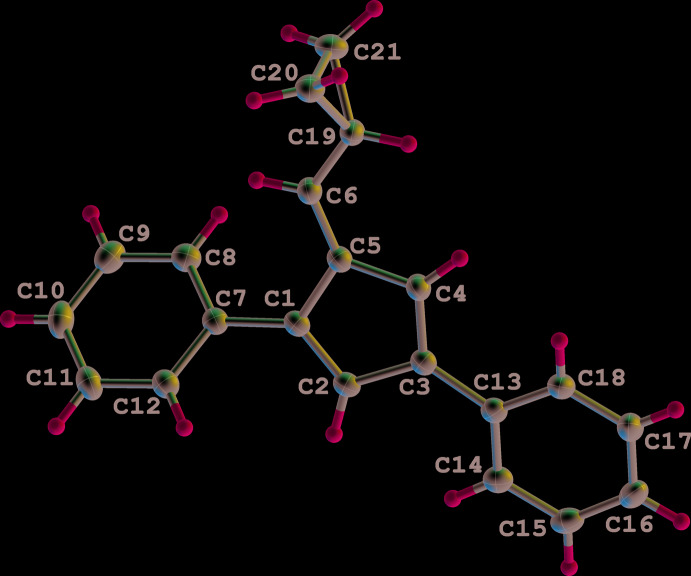
The mol­ecular structure of **1**. Displacement ellipsoids are shown at the 50% probability level.

**Figure 2:**
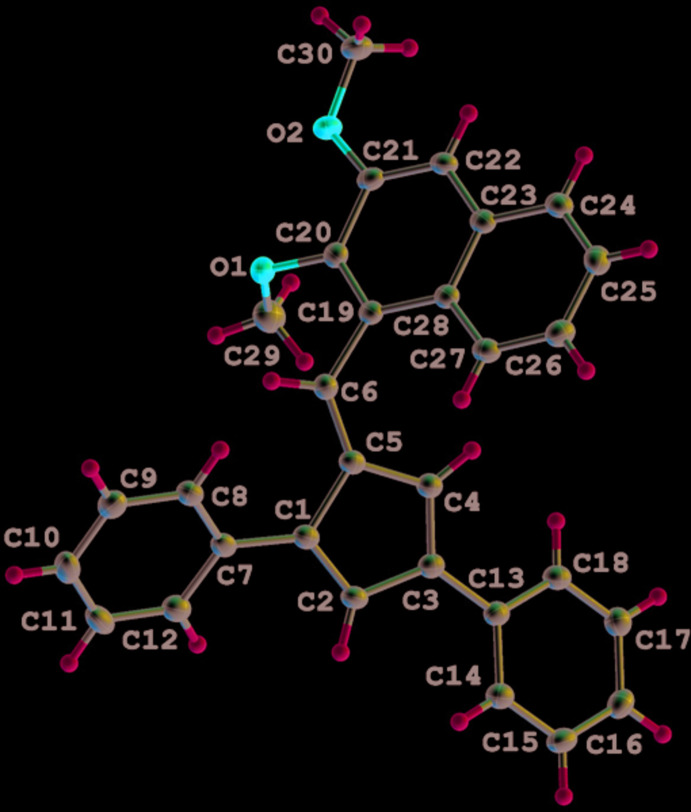
The mol­ecular structure of **2**. Displacement ellipsoids are shown at the 50% probability level.

Compounds **1** (Fig. 1 ▸) and **2** (Fig. 2 ▸) crystallize in the ortho­rhom­bic space groups *Pbca* and *P*2_1_2_1_2_1_, respectively. Both fulvenes crystallize with one mol­ecule per asymmetric unit (*Z*′ = 1), exhibit the expected alternating short–long bond lengths within the fulvene core and display very similar bond lengths and angles (Table 1 ▸). Similar tilt angles of the phenyl substituents from the plane of the fulvene ring are also observed for **1** [1-Ph, 44.88 (4)°; 3-Ph 13.34 (4)°] and **2** [1-Ph, 30.82 (7)°; 3-Ph 17.19 (7)°]. Surprisingly, the rotation of the 6-substituent from the cyclo­penta­dienyl core is greater for fulvene **1** [87.20 (6)°], than for the larger 2,3-di­meth­oxy­naphthalene substituent in fulvene **2**, [55.63 (5)°].

## Supra­molecular features   

**Figure 3:**
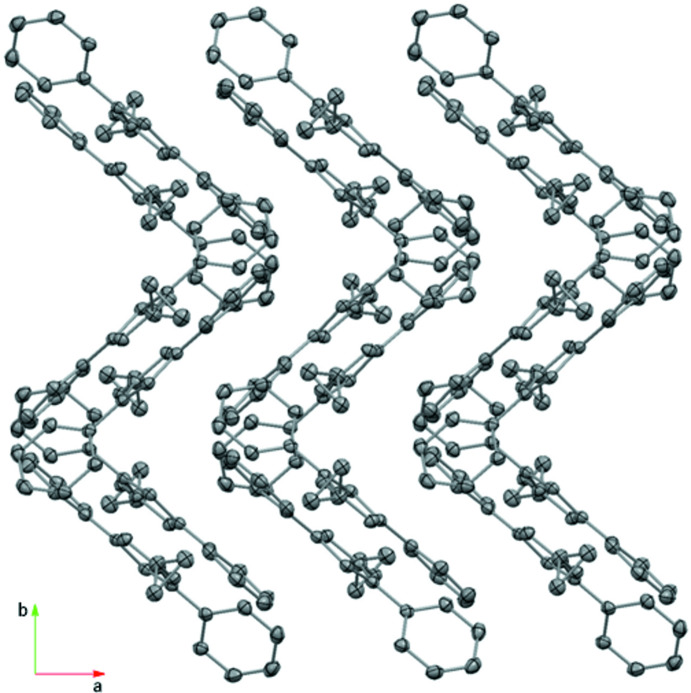
The packing of 6-cyclo­propyl-1,3-di­phenyl­fulvene, **1**, viewed along the *c*-axis direction. Hydrogen atoms are omitted for clarity.

Fulvene **1** packs side by side along the *a*-axis direction with mol­ecules oriented in such a way that the 6-cyclo­propyl groups are sandwiched between the 1-Ph and 3-Ph rings of adjacent fulvene mol­ecules. The closest contacts caused by this stacking sequence in the *a*-axis direction are between the 1-Ph ring atom H9 and the exocyclic C6 atom of an adjacent fulvene (C—H⋯C = 2.90 Å). Other C—H⋯C contacts (C12⋯H19 = 2.83, C2⋯H10 = 2.85, C14⋯H20*B* = 2.85, C11⋯H19 = 2.86 Å) lead to the formation of a network that results in sets of zigzag chains running perpendicular to the *a*-axis direction and that extend in the direction parallel to the *bc* plane (Fig. 3 ▸).

**Figure 4:**
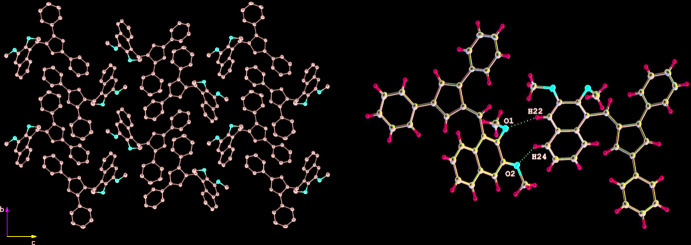
The packing of 6-(2,3-di­meth­oxy­naphth­yl)-1,3-di­phenyl­fulvene, **2**, viewed along the *a*-axis direction (left) and C—H⋯O inter­actions (right). Hydrogen atoms are omitted for clarity (left).

Fulvene **2** packs so that the 1-Ph groups are oriented towards the space between the 2,3-di­meth­oxy­naphthyl groups and the 3-Ph rings of adjacent fulvene mol­ecules along the *b*-axis direction. A view down the *a* axis (Fig. 4 ▸) reveals layers of inter­laced 2,3-di­meth­oxy­naphthyl groups (H, head) oriented H–H and separated from layers of inter­laced 1,3-di­phenyl­fulvene groups (T, tail) oriented T–T, with the layers running perpendicular to the *c*-axis direction and producing a layer sequence of H–H–T–T along the *c*-axis direction. In the H–H layers, short inter­molecular contacts of the C—H⋯O type (O1⋯H22 = 2.50 and O2⋯H24 = 2.53 Å; Table 2 ▸) occur between adjacent 2,3-di­meth­oxy­naphthyl groups running along the *b*-axis direction (Fig. 4 ▸). The meth­oxy groups apparently prevent the naphthyl rings from forming any π–π stacking inter­actions, with the angle between the mean planes of the 2,3-di­meth­oxy­naphthyl groups oriented at 124.37 (5)° at least partially enforced by the C—H⋯O inter­actions.

## Database survey   

A survey of the December 2019 release of the Cambridge Structural Database, with updates through November 2019, was made using the program *Conquest* (Groom *et al.*, 2016 ▸). A search for 1,3-diphenyl-6-substituted fulvenes yielded 88 results. The bond lengths and angles in **1** and **2** are consistent with those in the previously reported literature.

## Synthesis and crystallization   

Each compound was prepared according to the established literature procedure (Peloquin *et al.*, 2012 ▸; Godman *et al.*, 2016 ▸).

**6-(Cyclo­prop­yl)-1,3-diphenylfulvene, 1**. Orange crystals suitable for single crystal X-ray diffraction were obtained from petroleum ether by slow evaporation.

**6-(2,3-Di­meth­oxy­napth­yl)-1,3-diphenylfulvene**, **2**. Red crystals suitable for single crystal X-ray diffraction were obtained from slow diffusion of petroleum ether into a DCM solution.

## Refinement   

Crystal data, data collection and structure refinement details are summarized in Table 3 ▸. H atoms were placed in calculated positions (0.95–1.00 Å) and refined as riding with *U*_iso_(H) = 1.2*U*_eq_(C) or 1.5*U*_eq_(C-meth­yl).

## Supplementary Material

Crystal structure: contains datablock(s) 1, 2. DOI: 10.1107/S2056989020006441/yk2129sup1.cif

Structure factors: contains datablock(s) 1. DOI: 10.1107/S2056989020006441/yk21291sup2.hkl

Supporting information file. DOI: 10.1107/S2056989020006441/yk21291sup4.cdx

Structure factors: contains datablock(s) 2. DOI: 10.1107/S2056989020006441/yk21292sup3.hkl

Supporting information file. DOI: 10.1107/S2056989020006441/yk21291sup5.cml

Supporting information file. DOI: 10.1107/S2056989020006441/yk21292sup6.cml

CCDC references: 2003795, 2003794

Additional supporting information:  crystallographic information; 3D view; checkCIF report

## Figures and Tables

**Table 1:** Selected bond distances and angles (Å) for fulvenes **1** and **2**

	**1**	**2**
C1—C2	1.3577 (13)	1.357 (2)
C1—C5	1.4774 (13)	1.489 (2)
C2—C3	1.4699 (13)	1.475 (2)
C3—C4	1.3621 (13)	1.354 (2)
C4—C5	1.4538 (13)	1.455 (2)
C5—C6	1.3520 (13)	1.351 (2)
C1—C7	1.4721 (13)	1.475 (2)
C3—13	1.4704 (13)	1.469 (2)
C6—C19	1.4570 (13)	1.476 (2)
		
C2—C1—C5	107.36 (8)	106.64 (15)
C1—C2—C3	109.71 (8)	110.03 (15)
C2—C3—C4	107.70 (8)	107.90 (15)
C3—C4—C5	109.12 (8)	109.07 (16)
C4—C5—C1	106.08 (8)	106.34 (14)
C4—C5—C6	126.91 (9)	126.27 (16)
C1—C5—C6	126.72 (9)	127.15 (16)

**Table 2:** Hydrogen-bond geometry (Å, °) for **2** [Chem scheme1]

*D*—H⋯*A*	*D*—H	H⋯*A*	*D*⋯*A*	*D*—H⋯*A*
C22—H22⋯O1^i^	0.95	2.50	3.429 (2)	164
C24—H24⋯O2^i^	0.95	2.53	3.241 (2)	131

Symmetry code: (i) 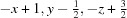.

**Table 3:** Experimental details

	**1**	**2**
Crystal data		
Chemical formula	C_21_H_18_	C_30_H_24_O_2_
*M*_r_	270.35	416.49
Crystal system, space group	Orthorhombic, *P* *b* *c* *a*	Orthorhombic, *P*2_1_2_1_2_1_
Temperature (K)	100	100
*a*, *b*, *c* (Å)	12.9844 (2), 11.9583 (1), 19.3729 (2)	7.3431 (1), 11.5468 (1), 25.7555 (3)
*V* (Å^3^)	3008.06 (6)	2183.79 (4)
*Z*	8	4
Radiation type	Mo *K*α	Mo *K*α
μ (mm^−1^)	0.07	0.08
Crystal size (mm)	0.38 × 0.25 × 0.15	0.27 × 0.15 × 0.13

Data collection		
Diffractometer	XtaLAB Synergy, Single source at offset/far, HyPix3000	XtaLAB Synergy, Single source at offset/far, HyPix3000
Absorption correction	Empirical (using intensity measurements) (*CrysAlis PRO*; Rigaku OD, 2019 ▸)	Empirical (using intensity measurements) (*CrysAlis PRO*; Rigaku OD, 2019 ▸)
*T*_min_, *T*_max_	0.404, 1.000	0.735, 1.000
No. of measured, independent and observed [*I* > 2σ(*I*)] reflections	74031, 3353, 2967	54562, 4798, 4437
*R*_int_	0.030	0.036
(sin θ/λ)_max_ (Å^−1^)	0.648	0.647

Refinement		
*R*[*F* ^2^ > 2σ(*F* ^2^)], *wR*(*F* ^2^), *S*	0.036, 0.094, 1.08	0.034, 0.082, 1.04
No. of reflections	3353	4798
No. of parameters	190	291
H-atom treatment	H-atom parameters constrained	H-atom parameters constrained
Δρ_max_, Δρ_min_ (e Å^−3^)	0.20, −0.19	0.16, −0.19
Absolute structure	–	Flack *x* determined using 1774 quotients [(*I* ^+^)−(*I* ^−^)]/[(*I* ^+^)+(*I* ^−^)] (Parsons *et al.*, 2013 ▸)
Absolute structure parameter	–	−0.3 (4)

Computer programs: *CrysAlis PRO* (Rigaku OD, 2019 ▸), *SHELXT* (Sheldrick, 2015*a* ▸), *SHELXL* (Sheldrick, 2015*b* ▸) and *OLEX2* (Dolomanov *et al.*, 2009 ▸).

## supplementary crystallographic information

### 5-(Cyclopropylmethylidene)-1,3-diphenylcyclopenta-1,3-diene (1) Crystal data

**Table:**

C_21_H_18_	*D*_x_ = 1.194 Mg m^−^^3^
*M**_r_* = 270.35	Mo *K*α radiation, λ = 0.71073 Å
Orthorhombic, *P**b**c**a*	Cell parameters from 50470 reflections
*a* = 12.9844 (2) Å	θ = 2.1–27.4°
*b* = 11.9583 (1) Å	µ = 0.07 mm^−^^1^
*c* = 19.3729 (2) Å	*T* = 100 K
*V* = 3008.06 (6) Å^3^	Block, orange
*Z* = 8	0.38 × 0.25 × 0.15 mm
*F*(000) = 1152	

### 5-(Cyclopropylmethylidene)-1,3-diphenylcyclopenta-1,3-diene (1) Data collection

**Table:**

XtaLAB Synergy, Single source at offset/far, HyPix3000 diffractometer	3353 independent reflections
Radiation source: micro-focus sealed X-ray tube, PhotonJet (Mo) X-ray Source	2967 reflections with *I* > 2σ(*I*)
Mirror monochromator	*R*_int_ = 0.030
Detector resolution: 10.0000 pixels mm^-1^	θ_max_ = 27.4°, θ_min_ = 2.1°
ω scans	*h* = −16→16
Absorption correction: empirical (using intensity measurements) (CrysAlisPro; Rigaku OD, 2019)	*k* = −15→15
*T*_min_ = 0.404, *T*_max_ = 1.000	*l* = −24→24
74031 measured reflections	

### 5-(Cyclopropylmethylidene)-1,3-diphenylcyclopenta-1,3-diene (1) Refinement

**Table:**

Refinement on *F*^2^	Primary atom site location: dual
Least-squares matrix: full	Hydrogen site location: inferred from neighbouring sites
*R*[*F*^2^ > 2σ(*F*^2^)] = 0.036	H-atom parameters constrained
*wR*(*F*^2^) = 0.094	*w* = 1/[σ^2^(*F*_o_^2^) + (0.045*P*)^2^ + 0.7758*P*] where *P* = (*F*_o_^2^ + 2*F*_c_^2^)/3
*S* = 1.08	(Δ/σ)_max_ < 0.001
3353 reflections	Δρ_max_ = 0.20 e Å^−^^3^
190 parameters	Δρ_min_ = −0.19 e Å^−^^3^
0 restraints	

### 5-(Cyclopropylmethylidene)-1,3-diphenylcyclopenta-1,3-diene (1) Special details

**Table:**

Geometry. All esds (except the esd in the dihedral angle between two l.s. planes) are estimated using the full covariance matrix. The cell esds are taken into account individually in the estimation of esds in distances, angles and torsion angles; correlations between esds in cell parameters are only used when they are defined by crystal symmetry. An approximate (isotropic) treatment of cell esds is used for estimating esds involving l.s. planes.

### 5-(Cyclopropylmethylidene)-1,3-diphenylcyclopenta-1,3-diene (1) Fractional atomic coordinates and isotropic or equivalent isotropic displacement parameters (Å^2^)

**Table:**

	*x*	*y*	*z*	*U*_iso_*/*U*_eq_	
C1	0.44298 (7)	0.64490 (8)	0.32819 (5)	0.0198 (2)	
C2	0.50655 (7)	0.60105 (8)	0.37681 (5)	0.0203 (2)	
H2	0.5051	0.6191	0.4246	0.024*	
C3	0.57790 (7)	0.52136 (8)	0.34441 (5)	0.0204 (2)	
C4	0.55715 (7)	0.51958 (8)	0.27550 (5)	0.0227 (2)	
H4	0.5918	0.4750	0.2422	0.027*	
C5	0.47342 (7)	0.59679 (8)	0.26098 (5)	0.0216 (2)	
C6	0.43760 (7)	0.62688 (8)	0.19814 (5)	0.0224 (2)	
H6	0.3823	0.6788	0.1965	0.027*	
C7	0.35860 (7)	0.72504 (8)	0.33954 (5)	0.0207 (2)	
C8	0.26145 (8)	0.70904 (9)	0.30946 (5)	0.0249 (2)	
H8	0.2502	0.6466	0.2801	0.030*	
C9	0.18165 (8)	0.78343 (9)	0.32221 (5)	0.0293 (2)	
H9	0.1163	0.7718	0.3013	0.035*	
C10	0.19672 (8)	0.87480 (9)	0.36532 (6)	0.0316 (2)	
H10	0.1422	0.9260	0.3737	0.038*	
C11	0.29212 (8)	0.89076 (9)	0.39608 (6)	0.0298 (2)	
H11	0.3026	0.9526	0.4260	0.036*	
C12	0.37234 (8)	0.81683 (8)	0.38334 (5)	0.0242 (2)	
H12	0.4373	0.8287	0.4046	0.029*	
C13	0.65376 (7)	0.45139 (8)	0.38061 (5)	0.0214 (2)	
C14	0.65376 (7)	0.44071 (8)	0.45261 (5)	0.0240 (2)	
H14	0.6078	0.4848	0.4794	0.029*	
C15	0.72009 (8)	0.36649 (8)	0.48540 (5)	0.0278 (2)	
H15	0.7187	0.3597	0.5343	0.033*	
C16	0.78805 (9)	0.30253 (9)	0.44717 (6)	0.0311 (2)	
H16	0.8319	0.2503	0.4696	0.037*	
C17	0.79200 (9)	0.31494 (9)	0.37585 (6)	0.0318 (2)	
H17	0.8400	0.2727	0.3495	0.038*	
C18	0.72593 (8)	0.38887 (8)	0.34322 (5)	0.0269 (2)	
H18	0.7296	0.3974	0.2945	0.032*	
C19	0.47766 (8)	0.58544 (8)	0.13271 (5)	0.0245 (2)	
H19	0.5338	0.5284	0.1360	0.029*	
C20	0.48463 (8)	0.66600 (9)	0.07225 (5)	0.0268 (2)	
H20A	0.4598	0.7434	0.0795	0.032*	
H20B	0.5447	0.6592	0.0411	0.032*	
C21	0.40700 (9)	0.57559 (9)	0.07072 (5)	0.0298 (2)	
H21A	0.4188	0.5125	0.0386	0.036*	
H21B	0.3339	0.5968	0.0769	0.036*	

### 5-(Cyclopropylmethylidene)-1,3-diphenylcyclopenta-1,3-diene (1) Atomic displacement parameters (Å^2^)

**Table:**

	*U*^11^	*U*^22^	*U*^33^	*U*^12^	*U*^13^	*U*^23^
C1	0.0211 (4)	0.0180 (4)	0.0203 (4)	−0.0038 (3)	0.0031 (3)	−0.0006 (3)
C2	0.0216 (4)	0.0199 (4)	0.0194 (4)	−0.0045 (4)	0.0023 (3)	−0.0010 (3)
C3	0.0212 (4)	0.0181 (4)	0.0218 (5)	−0.0036 (4)	0.0017 (4)	0.0002 (3)
C4	0.0256 (5)	0.0218 (5)	0.0208 (5)	0.0028 (4)	0.0020 (4)	−0.0006 (4)
C5	0.0236 (5)	0.0202 (4)	0.0209 (5)	−0.0002 (4)	0.0021 (4)	−0.0007 (4)
C6	0.0236 (5)	0.0213 (5)	0.0223 (5)	0.0019 (4)	0.0016 (4)	0.0001 (4)
C7	0.0232 (5)	0.0203 (4)	0.0187 (4)	−0.0013 (4)	0.0045 (4)	0.0026 (3)
C8	0.0246 (5)	0.0271 (5)	0.0230 (5)	−0.0025 (4)	0.0035 (4)	0.0011 (4)
C9	0.0225 (5)	0.0363 (6)	0.0291 (5)	0.0013 (4)	0.0040 (4)	0.0068 (4)
C10	0.0300 (5)	0.0297 (5)	0.0350 (6)	0.0086 (4)	0.0107 (4)	0.0063 (4)
C11	0.0359 (6)	0.0217 (5)	0.0318 (5)	0.0025 (4)	0.0073 (4)	−0.0014 (4)
C12	0.0267 (5)	0.0211 (5)	0.0248 (5)	−0.0016 (4)	0.0026 (4)	0.0006 (4)
C13	0.0221 (4)	0.0184 (4)	0.0237 (5)	−0.0039 (4)	−0.0019 (4)	−0.0003 (4)
C14	0.0234 (5)	0.0243 (5)	0.0244 (5)	−0.0057 (4)	−0.0002 (4)	0.0007 (4)
C15	0.0312 (5)	0.0272 (5)	0.0252 (5)	−0.0087 (4)	−0.0065 (4)	0.0046 (4)
C16	0.0321 (5)	0.0248 (5)	0.0366 (6)	−0.0004 (4)	−0.0138 (5)	0.0019 (4)
C17	0.0314 (6)	0.0292 (5)	0.0348 (6)	0.0074 (4)	−0.0080 (4)	−0.0071 (4)
C18	0.0290 (5)	0.0277 (5)	0.0240 (5)	0.0028 (4)	−0.0036 (4)	−0.0037 (4)
C19	0.0285 (5)	0.0250 (5)	0.0199 (5)	0.0059 (4)	−0.0001 (4)	−0.0002 (4)
C20	0.0300 (5)	0.0298 (5)	0.0207 (5)	0.0005 (4)	0.0025 (4)	0.0014 (4)
C21	0.0327 (5)	0.0344 (6)	0.0223 (5)	−0.0031 (4)	−0.0024 (4)	−0.0032 (4)

### 5-(Cyclopropylmethylidene)-1,3-diphenylcyclopenta-1,3-diene (1) Geometric parameters (Å, º)

**Table:**

C1—C2	1.3577 (13)	C11—C12	1.3882 (14)
C1—C5	1.4774 (13)	C12—H12	0.9500
C1—C7	1.4721 (13)	C13—C14	1.4008 (14)
C2—H2	0.9500	C13—C18	1.4006 (14)
C2—C3	1.4699 (13)	C14—H14	0.9500
C3—C4	1.3621 (13)	C14—C15	1.3903 (14)
C3—C13	1.4704 (13)	C15—H15	0.9500
C4—H4	0.9500	C15—C16	1.3829 (16)
C4—C5	1.4538 (13)	C16—H16	0.9500
C5—C6	1.3520 (13)	C16—C17	1.3905 (16)
C6—H6	0.9500	C17—H17	0.9500
C6—C19	1.4570 (13)	C17—C18	1.3847 (14)
C7—C8	1.4027 (14)	C18—H18	0.9500
C7—C12	1.3988 (13)	C19—H19	1.0000
C8—H8	0.9500	C19—C20	1.5193 (14)
C8—C9	1.3879 (14)	C19—C21	1.5159 (14)
C9—H9	0.9500	C20—H20A	0.9900
C9—C10	1.3891 (16)	C20—H20B	0.9900
C10—H10	0.9500	C20—C21	1.4784 (15)
C10—C11	1.3878 (16)	C21—H21A	0.9900
C11—H11	0.9500	C21—H21B	0.9900
			
C2—C1—C5	107.36 (8)	C14—C13—C3	121.79 (9)
C2—C1—C7	126.89 (9)	C18—C13—C3	120.35 (9)
C7—C1—C5	125.75 (8)	C18—C13—C14	117.80 (9)
C1—C2—H2	125.1	C13—C14—H14	119.6
C1—C2—C3	109.71 (8)	C15—C14—C13	120.88 (9)
C3—C2—H2	125.1	C15—C14—H14	119.6
C2—C3—C13	125.97 (8)	C14—C15—H15	119.9
C4—C3—C2	107.70 (8)	C16—C15—C14	120.24 (10)
C4—C3—C13	126.23 (9)	C16—C15—H15	119.9
C3—C4—H4	125.4	C15—C16—H16	120.1
C3—C4—C5	109.12 (8)	C15—C16—C17	119.78 (10)
C5—C4—H4	125.4	C17—C16—H16	120.1
C4—C5—C1	106.08 (8)	C16—C17—H17	120.0
C6—C5—C1	126.72 (9)	C18—C17—C16	119.93 (10)
C6—C5—C4	126.91 (9)	C18—C17—H17	120.0
C5—C6—H6	117.6	C13—C18—H18	119.4
C5—C6—C19	124.75 (9)	C17—C18—C13	121.28 (10)
C19—C6—H6	117.6	C17—C18—H18	119.4
C8—C7—C1	121.23 (9)	C6—C19—H19	115.9
C12—C7—C1	120.44 (9)	C6—C19—C20	118.44 (9)
C12—C7—C8	118.28 (9)	C6—C19—C21	119.97 (9)
C7—C8—H8	119.7	C20—C19—H19	115.9
C9—C8—C7	120.66 (10)	C21—C19—H19	115.9
C9—C8—H8	119.7	C21—C19—C20	58.30 (7)
C8—C9—H9	119.8	C19—C20—H20A	117.7
C8—C9—C10	120.40 (10)	C19—C20—H20B	117.7
C10—C9—H9	119.8	H20A—C20—H20B	114.8
C9—C10—H10	120.3	C21—C20—C19	60.74 (7)
C11—C10—C9	119.48 (10)	C21—C20—H20A	117.7
C11—C10—H10	120.3	C21—C20—H20B	117.7
C10—C11—H11	119.8	C19—C21—H21A	117.7
C10—C11—C12	120.38 (10)	C19—C21—H21B	117.7
C12—C11—H11	119.8	C20—C21—C19	60.96 (7)
C7—C12—H12	119.6	C20—C21—H21A	117.7
C11—C12—C7	120.79 (10)	C20—C21—H21B	117.7
C11—C12—H12	119.6	H21A—C21—H21B	114.8

### 5-[(3,4-Dimethoxynaphthalen-2-yl)methylidene]-1,3-diphenylcyclopenta-1,3-diene (2) Crystal data

**Table:**

C_30_H_24_O_2_	*D*_x_ = 1.267 Mg m^−^^3^
*M**_r_* = 416.49	Mo *K*α radiation, λ = 0.71073 Å
Orthorhombic, *P*2_1_2_1_2_1_	Cell parameters from 35583 reflections
*a* = 7.3431 (1) Å	θ = 1.9–27.3°
*b* = 11.5468 (1) Å	µ = 0.08 mm^−^^1^
*c* = 25.7555 (3) Å	*T* = 100 K
*V* = 2183.79 (4) Å^3^	Block, red
*Z* = 4	0.27 × 0.15 × 0.13 mm
*F*(000) = 880	

### 5-[(3,4-Dimethoxynaphthalen-2-yl)methylidene]-1,3-diphenylcyclopenta-1,3-diene (2) Data collection

**Table:**

XtaLAB Synergy, Single source at offset/far, HyPix3000 diffractometer	4798 independent reflections
Radiation source: micro-focus sealed X-ray tube, PhotonJet (Mo) X-ray Source	4437 reflections with *I* > 2σ(*I*)
Mirror monochromator	*R*_int_ = 0.036
Detector resolution: 10.0000 pixels mm^-1^	θ_max_ = 27.4°, θ_min_ = 1.9°
ω scans	*h* = −9→9
Absorption correction: empirical (using intensity measurements) (CrysAlisPro; Rigaku OD, 2019)	*k* = −14→14
*T*_min_ = 0.735, *T*_max_ = 1.000	*l* = −32→32
54562 measured reflections	

### 5-[(3,4-Dimethoxynaphthalen-2-yl)methylidene]-1,3-diphenylcyclopenta-1,3-diene (2) Refinement

**Table:**

Refinement on *F*^2^	Hydrogen site location: inferred from neighbouring sites
Least-squares matrix: full	H-atom parameters constrained
*R*[*F*^2^ > 2σ(*F*^2^)] = 0.034	*w* = 1/[σ^2^(*F*_o_^2^) + (0.0415*P*)^2^ + 0.3848*P*] where *P* = (*F*_o_^2^ + 2*F*_c_^2^)/3
*wR*(*F*^2^) = 0.082	(Δ/σ)_max_ = 0.001
*S* = 1.04	Δρ_max_ = 0.16 e Å^−^^3^
4798 reflections	Δρ_min_ = −0.18 e Å^−^^3^
291 parameters	Absolute structure: Flack *x* determined using 1774 quotients [(*I*^+^)-(*I*^-^)]/[(*I*^+^)+(*I*^-^)] (Parsons *et al.*, 2013)
0 restraints	Absolute structure parameter: −0.3 (4)
Primary atom site location: dual	

### 5-[(3,4-Dimethoxynaphthalen-2-yl)methylidene]-1,3-diphenylcyclopenta-1,3-diene (2) Special details

**Table:**

Geometry. All esds (except the esd in the dihedral angle between two l.s. planes) are estimated using the full covariance matrix. The cell esds are taken into account individually in the estimation of esds in distances, angles and torsion angles; correlations between esds in cell parameters are only used when they are defined by crystal symmetry. An approximate (isotropic) treatment of cell esds is used for estimating esds involving l.s. planes.

### 5-[(3,4-Dimethoxynaphthalen-2-yl)methylidene]-1,3-diphenylcyclopenta-1,3-diene (2) Fractional atomic coordinates and isotropic or equivalent isotropic displacement parameters (Å^2^)

**Table:**

	*x*	*y*	*z*	*U*_iso_*/*U*_eq_	
O1	0.44518 (18)	0.62766 (11)	0.68257 (5)	0.0247 (3)	
O2	0.61050 (17)	0.49358 (11)	0.75201 (5)	0.0259 (3)	
C1	−0.0406 (2)	0.65397 (15)	0.55785 (7)	0.0198 (4)	
C2	−0.0799 (2)	0.59770 (15)	0.51299 (7)	0.0211 (4)	
H2	−0.1346	0.6322	0.4834	0.025*	
C3	−0.0254 (2)	0.47506 (15)	0.51698 (7)	0.0197 (4)	
C4	0.0428 (3)	0.45757 (16)	0.56522 (6)	0.0207 (4)	
H4	0.0855	0.3857	0.5784	0.025*	
C5	0.0400 (2)	0.56652 (15)	0.59360 (6)	0.0196 (4)	
C6	0.1117 (2)	0.58571 (15)	0.64113 (7)	0.0204 (4)	
H6	0.1098	0.6635	0.6531	0.024*	
C7	−0.0696 (2)	0.77839 (15)	0.56788 (7)	0.0201 (4)	
C8	−0.1120 (3)	0.82200 (15)	0.61722 (7)	0.0232 (4)	
H8	−0.1192	0.7705	0.6459	0.028*	
C9	−0.1436 (3)	0.93976 (16)	0.62481 (8)	0.0276 (4)	
H9	−0.1710	0.9678	0.6586	0.033*	
C10	−0.1352 (3)	1.01607 (17)	0.58346 (8)	0.0302 (4)	
H10	−0.1570	1.0963	0.5887	0.036*	
C11	−0.0945 (3)	0.97418 (17)	0.53435 (8)	0.0302 (4)	
H11	−0.0895	1.0260	0.5058	0.036*	
C12	−0.0612 (3)	0.85764 (16)	0.52664 (7)	0.0252 (4)	
H12	−0.0321	0.8307	0.4928	0.030*	
C13	−0.0383 (2)	0.38948 (15)	0.47494 (6)	0.0198 (4)	
C14	−0.1483 (3)	0.40870 (16)	0.43146 (7)	0.0240 (4)	
H14	−0.2167	0.4783	0.4289	0.029*	
C15	−0.1585 (3)	0.32715 (17)	0.39190 (7)	0.0274 (4)	
H15	−0.2351	0.3408	0.3628	0.033*	
C16	−0.0574 (3)	0.22612 (16)	0.39474 (7)	0.0252 (4)	
H16	−0.0638	0.1707	0.3675	0.030*	
C17	0.0532 (3)	0.20609 (16)	0.43754 (7)	0.0243 (4)	
H17	0.1233	0.1371	0.4395	0.029*	
C18	0.0617 (3)	0.28622 (16)	0.47734 (7)	0.0229 (4)	
H18	0.1363	0.2711	0.5067	0.028*	
C19	0.1926 (2)	0.49925 (15)	0.67648 (7)	0.0194 (4)	
C20	0.3601 (2)	0.52592 (15)	0.69714 (7)	0.0206 (4)	
C21	0.4485 (3)	0.45362 (15)	0.73404 (6)	0.0211 (4)	
C22	0.3672 (3)	0.35129 (15)	0.74809 (7)	0.0216 (4)	
H22	0.4269	0.3012	0.7719	0.026*	
C23	0.1953 (3)	0.31938 (15)	0.72749 (6)	0.0198 (4)	
C24	0.1143 (3)	0.21256 (16)	0.74162 (7)	0.0233 (4)	
H24	0.1763	0.1620	0.7647	0.028*	
C25	−0.0526 (3)	0.18134 (16)	0.72228 (7)	0.0254 (4)	
H25	−0.1043	0.1087	0.7314	0.030*	
C26	−0.1476 (3)	0.25666 (16)	0.68892 (7)	0.0250 (4)	
H26	−0.2644	0.2353	0.6762	0.030*	
C27	−0.0726 (2)	0.36052 (16)	0.67463 (6)	0.0222 (4)	
H27	−0.1393	0.4111	0.6526	0.027*	
C28	0.1027 (2)	0.39369 (15)	0.69210 (6)	0.0187 (4)	
C29	0.5376 (3)	0.61847 (19)	0.63345 (8)	0.0317 (4)	
H29A	0.4567	0.5819	0.6080	0.048*	
H29B	0.6476	0.5714	0.6376	0.048*	
H29C	0.5714	0.6960	0.6213	0.048*	
C30	0.6930 (3)	0.42942 (18)	0.79361 (8)	0.0297 (4)	
H30A	0.7212	0.3508	0.7817	0.044*	
H30B	0.6085	0.4256	0.8230	0.044*	
H30C	0.8055	0.4681	0.8045	0.044*	

### 5-[(3,4-Dimethoxynaphthalen-2-yl)methylidene]-1,3-diphenylcyclopenta-1,3-diene (2) Atomic displacement parameters (Å^2^)

**Table:**

	*U*^11^	*U*^22^	*U*^33^	*U*^12^	*U*^13^	*U*^23^
O1	0.0290 (7)	0.0200 (6)	0.0252 (6)	−0.0041 (6)	−0.0044 (6)	0.0013 (5)
O2	0.0258 (6)	0.0266 (7)	0.0251 (7)	−0.0008 (6)	−0.0095 (6)	0.0015 (5)
C1	0.0178 (8)	0.0220 (9)	0.0195 (8)	−0.0005 (7)	−0.0003 (7)	0.0025 (7)
C2	0.0220 (9)	0.0227 (9)	0.0186 (8)	0.0009 (8)	−0.0026 (7)	0.0029 (7)
C3	0.0175 (8)	0.0227 (9)	0.0189 (8)	−0.0013 (7)	0.0002 (7)	0.0006 (7)
C4	0.0225 (8)	0.0208 (9)	0.0187 (8)	0.0006 (7)	−0.0008 (7)	0.0008 (7)
C5	0.0182 (8)	0.0218 (9)	0.0188 (8)	−0.0006 (7)	0.0002 (7)	0.0022 (7)
C6	0.0214 (8)	0.0184 (8)	0.0212 (8)	0.0014 (7)	−0.0005 (7)	−0.0004 (7)
C7	0.0167 (8)	0.0206 (9)	0.0229 (9)	−0.0004 (7)	−0.0033 (7)	0.0032 (7)
C8	0.0244 (9)	0.0224 (9)	0.0226 (9)	0.0014 (8)	−0.0031 (7)	0.0023 (7)
C9	0.0281 (10)	0.0264 (10)	0.0283 (10)	0.0029 (8)	−0.0048 (8)	−0.0039 (8)
C10	0.0294 (10)	0.0193 (9)	0.0420 (11)	0.0030 (8)	−0.0075 (9)	0.0002 (8)
C11	0.0322 (10)	0.0247 (10)	0.0336 (10)	−0.0016 (9)	−0.0049 (9)	0.0108 (8)
C12	0.0250 (9)	0.0269 (10)	0.0239 (9)	−0.0012 (8)	−0.0017 (8)	0.0027 (7)
C13	0.0198 (8)	0.0220 (9)	0.0174 (8)	−0.0025 (7)	0.0010 (7)	0.0013 (7)
C14	0.0258 (9)	0.0248 (9)	0.0215 (9)	0.0000 (8)	−0.0017 (7)	0.0013 (7)
C15	0.0312 (10)	0.0309 (10)	0.0201 (9)	−0.0032 (8)	−0.0048 (8)	−0.0002 (8)
C16	0.0294 (10)	0.0253 (9)	0.0211 (9)	−0.0052 (8)	0.0031 (8)	−0.0043 (7)
C17	0.0251 (9)	0.0227 (9)	0.0252 (9)	0.0000 (8)	0.0026 (8)	−0.0002 (7)
C18	0.0249 (9)	0.0250 (9)	0.0189 (9)	0.0002 (8)	−0.0010 (7)	0.0015 (7)
C19	0.0240 (9)	0.0186 (9)	0.0155 (8)	0.0026 (7)	0.0000 (7)	−0.0027 (7)
C20	0.0251 (9)	0.0187 (9)	0.0179 (8)	0.0016 (8)	0.0005 (7)	−0.0025 (7)
C21	0.0228 (9)	0.0235 (9)	0.0170 (8)	0.0039 (7)	−0.0025 (7)	−0.0038 (7)
C22	0.0257 (9)	0.0227 (9)	0.0164 (8)	0.0047 (7)	−0.0025 (7)	0.0001 (7)
C23	0.0243 (9)	0.0207 (9)	0.0145 (8)	0.0031 (7)	0.0019 (7)	−0.0016 (7)
C24	0.0295 (10)	0.0232 (9)	0.0172 (8)	0.0031 (8)	0.0007 (7)	0.0007 (7)
C25	0.0317 (10)	0.0233 (9)	0.0210 (9)	−0.0039 (8)	0.0044 (8)	0.0011 (7)
C26	0.0236 (9)	0.0295 (10)	0.0219 (9)	−0.0030 (8)	0.0000 (8)	−0.0008 (7)
C27	0.0237 (9)	0.0248 (9)	0.0182 (8)	0.0027 (8)	0.0000 (7)	0.0005 (7)
C28	0.0219 (8)	0.0195 (8)	0.0148 (8)	0.0021 (7)	0.0016 (7)	−0.0019 (6)
C29	0.0296 (10)	0.0336 (11)	0.0320 (10)	−0.0052 (9)	0.0038 (9)	0.0054 (9)
C30	0.0308 (10)	0.0321 (11)	0.0261 (10)	0.0024 (9)	−0.0096 (9)	−0.0006 (8)

### 5-[(3,4-Dimethoxynaphthalen-2-yl)methylidene]-1,3-diphenylcyclopenta-1,3-diene (2) Geometric parameters (Å, º)

**Table:**

O1—C20	1.383 (2)	C14—C15	1.389 (3)
O1—C29	1.439 (2)	C15—H15	0.9500
O2—C21	1.357 (2)	C15—C16	1.385 (3)
O2—C30	1.436 (2)	C16—H16	0.9500
C1—C2	1.357 (2)	C16—C17	1.389 (3)
C1—C5	1.489 (2)	C17—H17	0.9500
C1—C7	1.475 (2)	C17—C18	1.382 (3)
C2—H2	0.9500	C18—H18	0.9500
C2—C3	1.475 (2)	C19—C20	1.375 (2)
C3—C4	1.354 (2)	C19—C28	1.443 (2)
C3—C13	1.469 (2)	C20—C21	1.422 (2)
C4—H4	0.9500	C21—C22	1.372 (3)
C4—C5	1.455 (2)	C22—H22	0.9500
C5—C6	1.351 (2)	C22—C23	1.418 (3)
C6—H6	0.9500	C23—C24	1.417 (3)
C6—C19	1.476 (2)	C23—C28	1.425 (2)
C7—C8	1.402 (2)	C24—H24	0.9500
C7—C12	1.403 (2)	C24—C25	1.371 (3)
C8—H8	0.9500	C25—H25	0.9500
C8—C9	1.393 (3)	C25—C26	1.408 (3)
C9—H9	0.9500	C26—H26	0.9500
C9—C10	1.384 (3)	C26—C27	1.370 (3)
C10—H10	0.9500	C27—H27	0.9500
C10—C11	1.387 (3)	C27—C28	1.417 (3)
C11—H11	0.9500	C29—H29A	0.9800
C11—C12	1.382 (3)	C29—H29B	0.9800
C12—H12	0.9500	C29—H29C	0.9800
C13—C14	1.398 (2)	C30—H30A	0.9800
C13—C18	1.402 (3)	C30—H30B	0.9800
C14—H14	0.9500	C30—H30C	0.9800
			
C20—O1—C29	112.90 (14)	C17—C16—H16	120.1
C21—O2—C30	116.64 (15)	C16—C17—H17	119.9
C2—C1—C5	106.64 (15)	C18—C17—C16	120.23 (18)
C2—C1—C7	125.79 (16)	C18—C17—H17	119.9
C7—C1—C5	127.55 (15)	C13—C18—H18	119.6
C1—C2—H2	125.0	C17—C18—C13	120.86 (17)
C1—C2—C3	110.03 (15)	C17—C18—H18	119.6
C3—C2—H2	125.0	C20—C19—C6	116.56 (16)
C4—C3—C2	107.90 (15)	C20—C19—C28	119.35 (16)
C4—C3—C13	126.85 (17)	C28—C19—C6	124.01 (16)
C13—C3—C2	125.22 (15)	O1—C20—C21	118.28 (16)
C3—C4—H4	125.5	C19—C20—O1	119.31 (16)
C3—C4—C5	109.07 (16)	C19—C20—C21	122.39 (17)
C5—C4—H4	125.5	O2—C21—C20	115.38 (16)
C4—C5—C1	106.34 (14)	O2—C21—C22	125.74 (16)
C6—C5—C1	127.15 (16)	C22—C21—C20	118.88 (17)
C6—C5—C4	126.27 (16)	C21—C22—H22	119.6
C5—C6—H6	116.4	C21—C22—C23	120.82 (16)
C5—C6—C19	127.22 (16)	C23—C22—H22	119.6
C19—C6—H6	116.4	C22—C23—C28	120.52 (16)
C8—C7—C1	122.72 (16)	C24—C23—C22	120.26 (17)
C8—C7—C12	117.49 (16)	C24—C23—C28	119.22 (17)
C12—C7—C1	119.75 (16)	C23—C24—H24	119.6
C7—C8—H8	119.5	C25—C24—C23	120.73 (18)
C9—C8—C7	120.99 (17)	C25—C24—H24	119.6
C9—C8—H8	119.5	C24—C25—H25	119.9
C8—C9—H9	119.8	C24—C25—C26	120.15 (18)
C10—C9—C8	120.40 (18)	C26—C25—H25	119.9
C10—C9—H9	119.8	C25—C26—H26	119.8
C9—C10—H10	120.3	C27—C26—C25	120.37 (18)
C9—C10—C11	119.30 (17)	C27—C26—H26	119.8
C11—C10—H10	120.3	C26—C27—H27	119.4
C10—C11—H11	119.7	C26—C27—C28	121.11 (17)
C12—C11—C10	120.59 (18)	C28—C27—H27	119.4
C12—C11—H11	119.7	C23—C28—C19	117.96 (16)
C7—C12—H12	119.4	C27—C28—C19	123.74 (16)
C11—C12—C7	121.22 (17)	C27—C28—C23	118.30 (16)
C11—C12—H12	119.4	O1—C29—H29A	109.5
C14—C13—C3	121.39 (16)	O1—C29—H29B	109.5
C14—C13—C18	118.22 (16)	O1—C29—H29C	109.5
C18—C13—C3	120.38 (16)	H29A—C29—H29B	109.5
C13—C14—H14	119.6	H29A—C29—H29C	109.5
C15—C14—C13	120.73 (17)	H29B—C29—H29C	109.5
C15—C14—H14	119.6	O2—C30—H30A	109.5
C14—C15—H15	119.9	O2—C30—H30B	109.5
C16—C15—C14	120.21 (18)	O2—C30—H30C	109.5
C16—C15—H15	119.9	H30A—C30—H30B	109.5
C15—C16—H16	120.1	H30A—C30—H30C	109.5
C15—C16—C17	119.73 (17)	H30B—C30—H30C	109.5

### 5-[(3,4-Dimethoxynaphthalen-2-yl)methylidene]-1,3-diphenylcyclopenta-1,3-diene (2) Hydrogen-bond geometry (Å, º)

**Table:**

*D*—H···*A*	*D*—H	H···*A*	*D*···*A*	*D*—H···*A*
C22—H22···O1^i^	0.95	2.50	3.429 (2)	164
C24—H24···O2^i^	0.95	2.53	3.241 (2)	131

Symmetry code: (i) −*x*+1, *y*−1/2, −*z*+3/2.
